# Effects of Channel Wall Twisting on the Mixing in a T-Shaped Micro-Channel

**DOI:** 10.3390/mi11010026

**Published:** 2019-12-24

**Authors:** Dong Jin Kang

**Affiliations:** School of Mechanical Engineering, Yeungnam University, Daehak-ro 280, Gyungsan 712-749, Korea; djkang@yu.ac.kr; Tel.: +82-53-810-2463

**Keywords:** T-shaped microchannel, degree of mixing, twisting angle

## Abstract

A new design scheme is proposed for twisting the walls of a microchannel, and its performance is demonstrated numerically. The numerical study was carried out for a T-shaped microchannel with twist angles in the range of 0 to 34π. The Reynolds number range was 0.15 to 6. The T-shaped microchannel consists of two inlet branches and an outlet branch. The mixing performance was analyzed in terms of the degree of mixing and relative mixing cost. All numerical results show that the twisting scheme is an effective way to enhance the mixing in a T-shaped microchannel. The mixing enhancement is realized by the swirling of two fluids in the cross section and is more prominent as the Reynolds number decreases. The twist angle was optimized to maximize the degree of mixing (DOM), which increases with the length of the outlet branch. The twist angle was also optimized in terms of the relative mixing cost (MC). The two optimum twisting angles are generally not coincident. The optimum twist angle shows a dependence on the length of the outlet branch but it is not affected much by the Reynolds number.

## 1. Introduction

Microscale fluid mixing is needed to homogenize reagents in many microfluidic systems, such as microreactors and micrototal analysis systems (μTASs). Applications include biological and chemical reactions, the dilution of drug solutions, and sequencing nucleic acids [1]. In these systems, the mixing is usually done in various types of microchannels. However, the fluid flows are extremely slow and have very low Reynolds numbers. Therefore, molecular diffusion is a major mechanism of mixing. It is very important to enhance the mixing for the design of microchannels [2,3].

The techniques to improve mixing in microchannels can be classified as passive, active, or combined techniques. One major difference is the usage of an external energy source other than the energy source that drives the flow. Active techniques use various types of external energy sources, such as electrokinetic [4], magneto-hydrodynamic [5], electroosmotic [3], ultrasound wave [6], and pulsed flow sources [7,8]. In contrast, passive techniques use the channel geometry or wall modifications to agitate or generate secondary flow in microchannels. Therefore, passive techniques are much easier to integrate into microfluidic systems. Combined methods involve both passive and active techniques. For example, Chen et al. [9] used a pulsatile flow through wavy channel walls, while Lim et al. [10] combined a periodic osmotic flow with geometry modification.

Passive techniques can be categorized into several groups according to how the channel is modified. Many passive techniques modify the channel wall of the outlet branch, which is the portion of a microchannel after the junction where the two fluids merge. Some examples use recessed grooves in the channel wall [11] and a herringbone wall [12]. The second type of technique involves building structures inside the channel, such as indentations and baffles [13,14], periodic geometric features [15], and a simple block in the junction [16].

The third type of technique involves rearranging the overall structure of the microchannel instead of using a straight microchannel. For example, Kashid et al. [17] studied five different generic microchannel designs with a focus on the region before the fluid merges. They tested five different layouts of inlet branches. Kockmann et al. [18] studied various mixer structures to obtain higher mixing in micromixers. Other examples are the AccoMix split-and-recombine technique by Panic et al. [19], the FAMOS multi-lamination micromixer by Keoschkerjan et al. [20], and the K-M collision micromixer by Schneider et al. [21]. These designs use complex elements such as multiple flow passages, 3-dimensional structures, and curved or non-straight channels.

Recently, a new concept of twisting the outlet branch has been studied to enhance the mixing in a microchannel. For example, Jafari et al. [22] studied a twisted channel with the Reynolds number ranging from 76.7 to 460.3. They coiled the outlet branch at a given twist angle, and showed that the mixing is enhanced with the twist angle. However, the required pressure load is also large therefore, the mixing cost becomes high. Sivashankar et al. [23] proposed a twisted 3D microfluidic mixer fabricated by a laser writing technique. They tested the twisted 3D microfluidic mixer in the range of volume flow rate from 1 μL/min to 1000 μL/min. The mixing efficiency is greatly reduced as the volume flow rate increases.

We proposed a new twisted channel geometry that is easily fabricated. We also characterized the mixing performance in a T-shaped microchannel. The design has a channel with twisted walls along the outlet branch. The mixing performance was studied numerically, and the performance was analyzed by calculating the degree of mixing, relative mixing cost and mixing energy cost. 

## 2. Microchannel with Twisted Channel Walls

**Figure 1:**
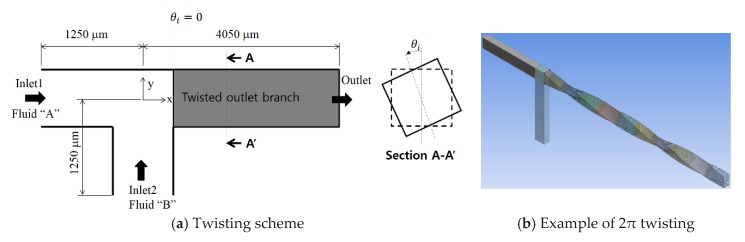
Diagram of a T-shaped micro-channel with twisting.

Figure 1 shows the layout of a T-shaped microchannel with three branches. All three branches have a rectangular cross section that is 200 μm high and 120 μm deep. Inlet 1 and inlet 2 are both 1250 μm long. The branch after the junction of the inlets is the outlet branch, which was varied from 2950 to 4050 μm long. The channel walls of the outlet branch are twisted, as shown in Figure 1a. The twisting angle was varied from 0 to 34π (17 revolutions). The shape of the cross section remains unchanged along the outlet branch. Figure 1b shows an example of 2π twisting (1 revolution).

For simplicity, we assume that the same aqueous solution flows into the two inlets. The fluid is assumed to have the properties found in many existing BioMEMS systems. Its diffusion constant is *D* = 10^−10^ m^2^s^−1^, and the kinematic viscosity of the fluid is *μ* = 10^−6^ m^2^s^−1^ at room temperature. This diffusion constant is typical of small proteins in an aqueous solution. The Schmidt (Sc) number is 10^4^ (the ratio of the kinetic viscosity and the mass diffusion of fluid).

## 3. Governing Equations and Computational Procedure

The fluid is assumed to be Newtonian and incompressible, and the equations of motion are the Navier–Stokes and continuity equations:

(1) $$\left(\vec{u}\cdot \nabla \right)\vec{u}=-\frac{1}{\rho }\nabla p+\nu \nabla^{2}\vec{u}$$

(2) $$\nabla \cdot \vec{u}=0.$$

where $\vec{u}$ is the velocity vector, *p* is the pressure, *ρ* is the mass density, and *ν* is the kinematic viscosity. The evolution of the concentration is computed from the advection diffusion equation:

(3) $$\left(\vec{u}\cdot \nabla \right)\phi =D\nabla^{2}\phi$$

where *D* is the diffusion constant, and *ϕ* is the local concentration or mass fraction of a given species. 

The governing equations (Equations (1)–(3)) were solved using the commercial software FLUENT 14.5 (ANSYS, Inc., Canonsburg, PA, USA). All of the convective terms in Equations (1) and (3) were approximated by the QUICK scheme (quadratic upstream interpolation for convective kinematics), which has third-order theoretical accuracy. A uniform velocity profile was assumed at the two inlets, while the outflow conditions were specified at the outlet. For example, the fluid velocity at the inlets is 1 (mm/s) for a Reynolds number of 0.3. All of the other walls were treated as no-slip walls. The mass fraction of the fluid was set to *ϕ* = 1 at inlet 1 and *ϕ* = 0 at inlet 2.

The mixing performance was evaluated by calculating the degree of mixing (DOM). The DOM defined by Glasgow et al. [5] is used in the following form:

(4) $$DOM=1-\frac{1}{\xi }\sqrt{\overset{n}{\underset{i=1}{\sum }}\frac{{\left(\phi_{i}-\xi \right)}^{2}}{n}\frac{u_{i}}{u_{mean}}}$$

where *u_i_* is the velocity in the *i*th cell, *u_mean_* is the mean velocity at the outlet of the microchannel, $\phi_{i}\;$ is the mass fraction in the *i*th cell, and *n* is the number of cells. ξ is specified as 0.5, which indicates equal mixing of the two solutions. Some researchers define the mixing performance (MP) in the following form [24,25]:

(5) $$MP=1-\frac{1}{\xi }\sqrt{\sum_{i=1}^{n}\frac{{\left(\phi_{i}-\xi \right)}^{2}}{n}}.$$

The relative mixing cost (MC) was also evaluated using the ratio of the mixing cost to the mixing cost obtained without any twist:

(6) $$MC=\frac{{\left(\frac{DOM}{\Delta p}\right)}_{twist}}{{\left(\frac{DOM}{\Delta p}\right)}_{no\;twist}}\;.$$

A smaller MC means that channel wall twisting is more effective. The fluid mixing, MF, is defined as follows:

(7) MF=1−2|0.5−ϕ|.

*MF* = 1 means that the fluid is completely mixed, while *MF* = 0 indicates an unmixed fluid of A or B. 

Some researchers calculate the mixing energy cost (MEC) by combining the pressure load and the MP to check the effectiveness of a micromixer [26,27,28]:

(8) MEC=∆pρumean2MPX100.

## 4. Validation of Numerical Study

**Figure 2:**
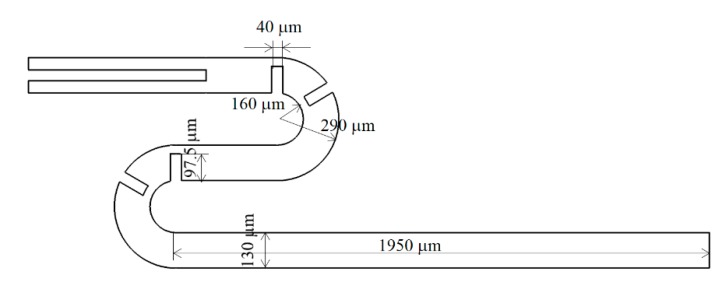
Diagram of a micro-channel experimented by Tsai et al. [25].

**Figure 3:**
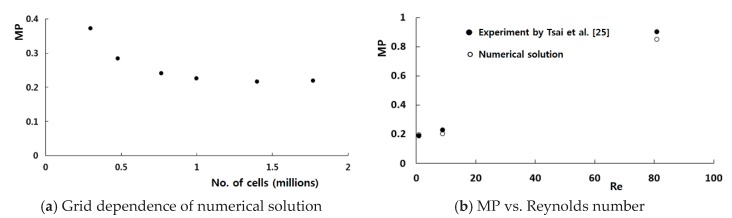
Validation of numerical solution. MP = Mixing performance.

To validate the present numerical approach, a micromixer experimented by Tsai et al. [25] was first simulated, and the results were compared with the corresponding experimental data. Figure 2 shows the schematic diagram of the micromixer; detailed size of the geometry is available in [25]. The length of the main channel is 5094 μm, and the cross section is a square of 130 μm. Therefore, the overall size is similar to that of the present microchannel. The computational domain was meshed by structured hexahedral cells. Before detailed simulations, a study was carried out to check the grid dependence of numerical solutions. Figure 3a shows the grid dependence of numerical solutions, and it becomes negligible when the number of cells is larger than about 1 million. The simulation results are also compared with the corresponding experimental data from Tsai et al. [25] for the Reynolds number from 1 to 81 in Figure 3b. The discrepancy between the numerical and experimental data is less than 10%, and it becomes smaller as the Reynolds number decreases. The discrepancy is attributed to several factors such as the numerical diffusion, experimental uncertainty, etc. However, they show the same behavior of the DOM vs. Reynolds number. The present numerical approach is used to evaluate the performance of the channel wall twisting design.

**Figure 4:**
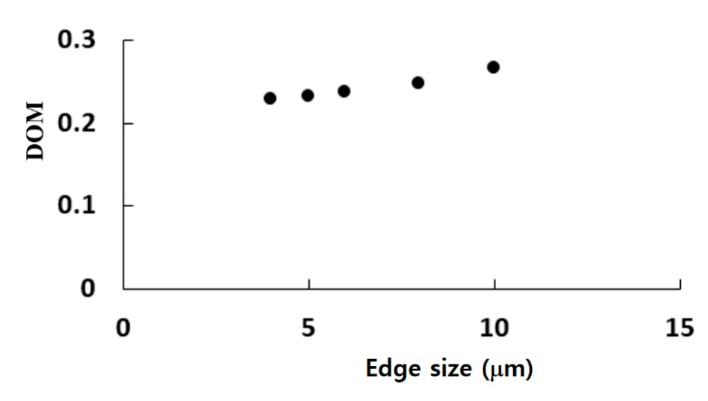
Variation of the DOM (degree of mixing) with the edge size of cells.

The twisted microchannel was meshed by structured hexahedral cells. All computational cells have equal size. The edge size of each cell was varied from 4 μm to 10 μm. A set of simulations was carried out to check the grid dependence of numerical solutions for the microchannel twisted by 360°. Figure 4 shows a variation of the calculated DOM with the edge size. The deviation of 5 μm and 6 μm solutions from that of 4 μm is 1.6% and 4%, respectively. Therefore, 5 μm is small enough to obtain grid independent solutions. 

An additional simulation was carried out for the baseline design without twisting. The simulated DOM at the section of *x* = 3 mm is 0.12, which is the same value reported by Goullet et al. [7]. The total mass flow rate at the outlet has an accuracy of 0.1%. 

## 5. Results and Discussion

**Figure 5:**
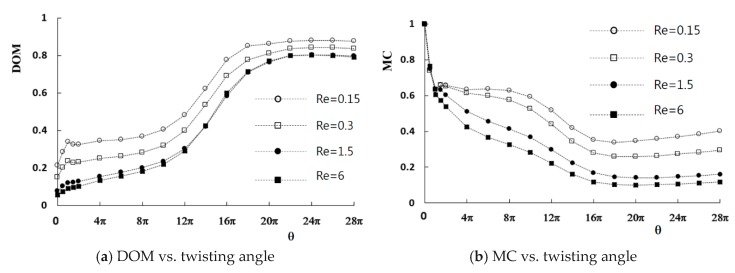
Variation of the DOM and MC (mixing cost) with the twist angle.

Computations were carried out for the given flow conditions to study how the twisting of the channel walls improves the mixing. The mean velocities at the two inlets are uniform, in the range from 0.5 mm/s to 20 mm/s, and the corresponding Reynolds number is 0.15 to 6. Figure 5 shows the computed DOM and the MC with respect to the twist angle *θ*. The DOM and MC were calculated at the outlet. The pressure difference was measured between the two inlets and outlet, and the larger value was used to compute the MC. The DOM shows a significant improvement as the twist angle increases, regardless of the Reynolds number. For example, the DOM with a twist angle of 24π (12 revolutions) is 0.843 for *Re* = 0.3, which is about four times larger than that obtained without twisting. 

The simulated mixing performance is compared, in terms of DOM, MP, and MEC with those from other mixing approaches in Table 1. Goullet et al. [7] used a pulsatile flow at the inlets. They have tested various pulsing conditions to enhance the mixing, and obtained the DOM of 0.78 for 5 Hz pulsatile flow with ribs. Wu et al. [24] used a converging-diverging meandering microchannel with semi-elliptical side walls. According to their result, the MP is about 0.65 when 10 modules of converging-diverging meandering section are inserted. Fang et al. [15] embedded periodic geometric features in the outlet branch, and obtained the MP of 0.519 for 10 geometric features embedded. Sheu et al. [25] studied a split and recombine design to enhance the mixing in a microchannel: the layout shown in Table 1 is two mixing segments of split and recombine. According to the results of their simulation, 17 mixing segments are required to obtain the mixing performance of 0.9, and it corresponds to the curved channel length of about 60 mm. The mixing energy cost was also summarized in Table 1. Each micromixer shows a different level of the mixing energy cost: a lower value means a more effective micromixer. Present results show a noticeable improvement from other mixing approaches for all of the volume flow rates. This comparison confirms that the present twisting of the outlet branch walls is a promising design scheme.

There is an optimum twist angle where the maximum DOM occurs. However, the optimum angle is almost independent of the Reynolds number. This implies that the mixing enhancement by the twisting of the channel walls is much larger than the mixing due to other mechanisms, such as the molecular diffusion and convective transport for the Reynolds numbers studied in this paper. The distribution of the DOM in Figure 5a shows that the effects of the Reynolds number decrease as the Reynolds number increases. This suggests that the twisting of the channel walls becomes a dominant mixing mechanism when the Reynolds number is greater than about 6.

**Figure 6:**
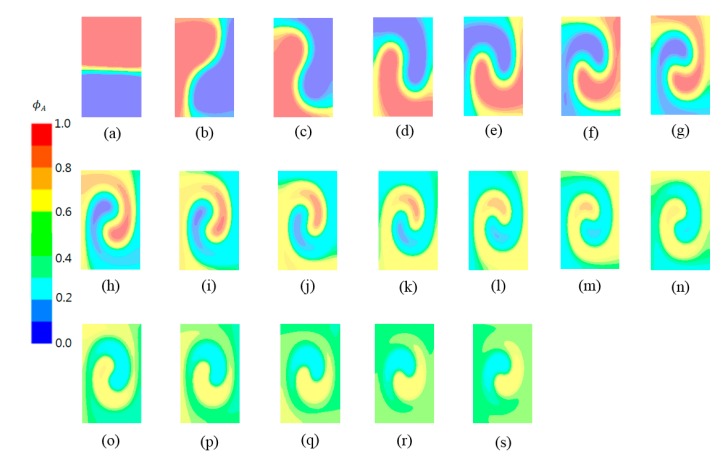
Mass fraction contours of the fluid A at several cross sections along the outlet branch: (**a**) *θ* = 0, (**b**) *θ* = π, (**c**) *θ* = 2π, (**d**) *θ* = 3π, (**e**) *θ* = 4π, (**f**) *θ* = 5π, (**g**) *θ* = 6π, (**h**) *θ* = 7π, (**i**) *θ* = 8π, (**j**) *θ* = 9π, (**k**) *θ* = 10π, (**l)** *θ* = 11π, (**m**) *θ* = 12π, (**n**) *θ* = 13π, (**o**) *θ* = 14π, (**p**) *θ* = 15π, (**q**) *θ* = 16π, (**r**) *θ* = 17π, (**s**) *θ* = 18π.

In contrast, the MC generally decreases as the twist angle increases. It also has an optimum value, as shown in Figure 5b. The optimum twist angle for the minimum MC is smaller than that of the maximum DOM. To examine how the twisting of channel walls improves the DOM, Figure 6 shows the mass fraction contours of the fluid A at several cross sections along the outlet branch. The results were obtained with a twist angle of 18π, where the minimum MC occurs. The Reynolds number is 0.3. The contours show that the fluids A and B rotate clockwise in the cross section as the channel walls twist in the counterclockwise direction. This swirling motion elongates the boundary between the fluids in the cross section, and the mixing is greatly enhanced along the boundary (green area in the figures).

The swirl motion is very slow compared with the rate of the channel wall twisting along the outlet branch. For example, fluid B (blue in Figure 6b) moves circumferentially by about 0.5π in comparison to Figure 6a when the cross section is twisted by π. Therefore, much greater twisting may hinder the swirling of fluids in the cross section. This suggests that there is an optimum twisting angle where the maximum DOM occurs.

**Figure 7:**
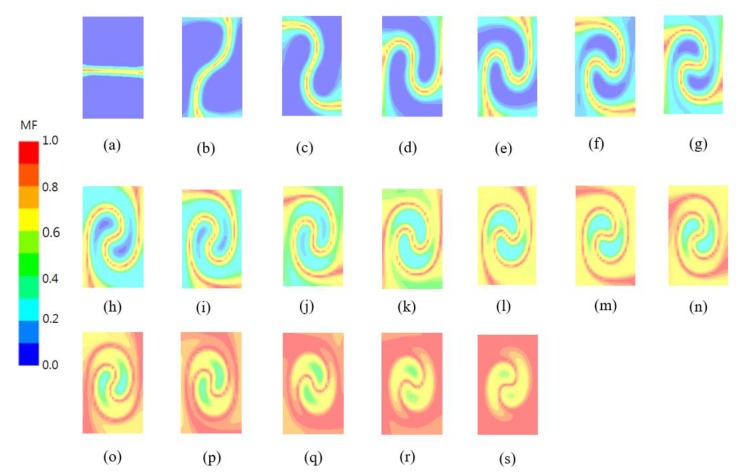
Distribution of the mixed fluid (MF) at several cross sections along the outlet branch: (**a**) *θ_i_* = 0, (**b**) *θ_i_* = π, (**c**) *θ_i_* = 2π, (**d**) *θ_i_* = 3π, (**e**) *θ_i_* = 4π, (**f**) *θ_i_* = 5π, (**g**) *θ_i_* = 6π, (**h**) *θ_i_* = 7π, (**i**) *θ_i_* = 8π, (**j**) *θ_i_* = 9π, (**k**) *θ_i_* = 10π, (**l)** *θ_i_* = 11π, (**m**) *θ_i_* = 12π, (**n**) *θ_i_* = 13π, (**o**) *θ_i_* = 14π, (**p**) *θ_i_* = 15π, (**q**) *θ_i_* = 16π, (**r**) *θ_i_* = 17π, (**s**) *θ_i_* = 18π.

Figure 7 shows the contours of the mixed fluid MF at the same planes as in Figure 6. The results confirm that the twisting causes vigorous mixing along the boundary. The red streak in the figures indicates the mixed fluid, which develops along the boundary. The length of the boundary increases with the twisting angle *θ_i_* of the cross section. The mixed fluid zone spreads out as the boundary impinges on the channel walls, which means that the channel walls slow down the swirling motion, and the mixed fluid spreads along the channel walls.

**Figure 8:**
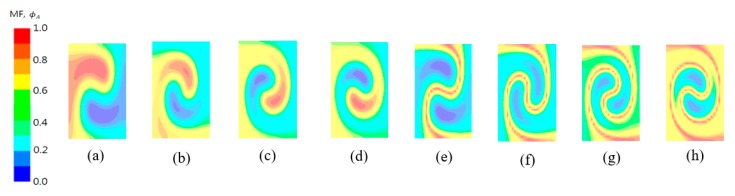
Mass fraction of fluid A and mixed fluid contours at the cross section of *θ_i_* = 8π for several twist angles: contours of *ϕ_A_* for (**a**) *θ* = 12π, (**b**) *θ* = 16π, (**c**) *θ* = 20π and (**d**) *θ* = 24π, contours of MF for (**e**) *θ* = 12π, (**f**) *θ* = 16π, (**g**) *θ* = 20π and (**h**) *θ* = 24π.

Figure 8 compares contours of the mass fraction of the fluid “A” and the mixed fluid MF at the cross section of *θ_i_* =8π for several twist angles. For a given length of the outlet branch, a larger twist angle results in a greater rate of twisting along the outlet branch. Figure 8a–d show how the twisting rate affects the swirl motion in the cross section. As the twisting rate increases, a stronger swirl motion is observed in the cross section with the same twist of *θ_i_* = 8π. A stronger swirl motion results in a longer boundary of the fluids A and B, as shown in Figure 8e–h. This eventually enhances the mixing of the two fluids along the boundary. 

**Figure 9:**
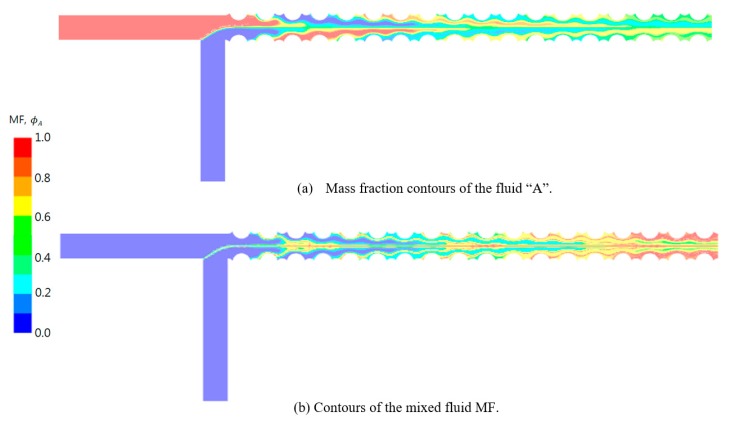
Contours of mass fraction of fluid A and the mixed fluid (MF) at the mid-section in the *z*-direction.

Figure 9 shows the mass fraction and the mixed fluid contours at the mid-section in the *z*-direction. The contours of the mass fraction show that the positions of fluids A and B move up and down successively as they flow downstream, which indicates the swirl motion in the cross section. The contours of the mixed fluid confirm that the mixing became greatly enhanced along the boundary between fluids A and B. The mixing occurs along the centerline from the junction of the fluids and is greatly enhanced near the channel wall as the fluids flow downstream. This enhancement is due to the swirl motion.

The DOM was optimized, and the corresponding twist angle remained almost constant for the range of Reynolds numbers studied. This suggests that the mixing enhancement mechanism is mostly affected by the twist angle. However, the swirl motion is very slow compared with the rate of the wall twisting, so excessive twisting may hinder the fluid swirling.

**Figure 10:**
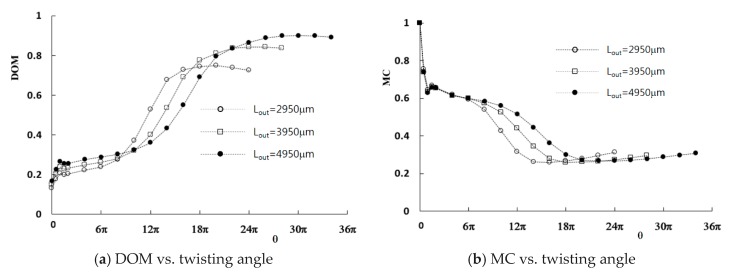
Effects of length of the outlet branch on mixing.

Figure 10 shows the effects of the length of the twisted outlet branch on the mixing. The DOM shows a strong dependence on the length of the outlet branch. The maximum DOM decreases as the length of the outlet branch decreases. The twist angle where the maximum of DOM occurs increases with the length of the outlet branch. For example, the angles are 10π, 12π, and 14π for *L_out_* = 2950, 3950, and 4950 μm, respectively. Similarly, the minimum of MC is obtained at a larger twist angle as the length of the outlet branch increases (8π, 9π, and 12π for *L_out_* = 2950, 3950, and 4950 μm, respectively). However, the ratio of the twist angle to the length of the outlet branch decreases as the length of the outlet branch increases. This means that greater twisting is required as the length of the outlet branch decreases.

## 6. Conclusions

This study numerically examined the effects of channel wall twisting on the mixing performance of a T-shaped microchannel. The performance was evaluated by examining the DOM and the MC. The channel walls were twisted continuously from the junction of the two inlet branches to the outlet, and the twist angle of the cross section increases continuously. This twisting scheme is easy to fabricate and very effective for improving the DOM.

The simulation results showed that the twisting significantly enhances the mixing due to the swirl motion of the fluids in the cross section along the outlet branch. In general, increasing the twist angle increases the swirl motion, which elongates the boundary between fluids A and B, and enhances the DOM. However, the swirl motion is very slow compared with the rate of the channel wall twisting along the outlet branch. Excessive twisting hinders the swirl and decreases the DOM, so there is an optimum twist angle where the maximum of DOM occurs. This optimum twist angle increases with the length of the outlet branch but is almost independent of the Reynolds number.

The twisting angle was also optimized in terms of the relative mixing cost. This twist angle is different from that where the maximum DOM occurs, and shows a dependency on the length of the outlet branch. Greater twisting is required as the length of the outlet branch decreases. As the Reynolds number increases, the twisting becomes dominant in mixing the fluids, and its effect on the DOM is limited. However, the relative mixing cost is improved further as the Reynolds number increases, which suggests that the twisting is a useful and passive design concept for a wide range of Reynolds numbers.

The present design scheme was shown to enhance the mixing performance in a microchannel for a wide range of the volume flow rate. It can be integrated into a variety of applications such as cell cultures, microfluidic filtration, and biochemistry analysis. 

## Figures and Tables

**Table 1:** Comparison of mixing performance.

Author	Cross Section/Channel Layout	Length of Outlet Branch (μm)	Width and Height (μm)	Volume Flow Rate (μL/min)	DOM/MP/MEC (Mixing Energy Cost)	Mixing Enhancement Mechanism
Present	Rectangle 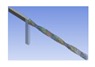	3950	200, 120	1.44	0.881/0.919/103.6	Wall twisting
				2.88	0.843/0.881/54.1	
				14.4	0.804/0.87/10.97	
				57.6	0.802/0.865/2.79	
Goullet et al. [7]	Rectangle 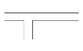	3950	200, 120	2.88	0.78/-/-	Pulsatile inlet flows
Wu et al. [24]	Rectangle 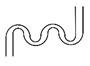	5094	120, 120	2.16	-/≈0.68/≈47.1	Converging-diverging meandering channel
				25.2	-/0.96/≈551	
Tsai et al. [25]	Square 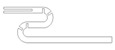	4255	130, 130	7.8	-/0.194/≈174.2	Radial baffle
				70.2	-/0.202/≈19.6	
				631.8	-/0.85/≈0.76	
Fang et al. [15]	Rectangle 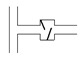	6000 (10 periods)	200, 300	20	-/0.519/-	Insertion of periodic features
		15,000 (28 periods)			-/0.794/-	
Sheu et al. [25]	Rectangle 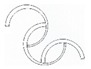	~8010 (2 mixing segments)	100, 100	60 (different aspect ratio)	-/0.405/4.61	Split and recombine
					-/0.292/4.5	
					-/0.295/4.17	
					-/0.340/6.02	
		60,000 (17 mixing segments)		60	-/0.9/-	
